# Integrating health promotion into biology education: effects of classroom and extracurricular interventions in rural adolescents

**DOI:** 10.3389/fnut.2026.1813900

**Published:** 2026-05-28

**Authors:** Jie Chen, Ruiping Dang, Hua Tian

**Affiliations:** 1 School of Marxism, Xinyang Normal University, Xinyang, Henan, China; 2 College of Life Science, Xinyang Normal University, Xinyang, Henan, China; 3 College of Tea and Food Science, Xinyang Normal University, Xinyang, Henan, China

**Keywords:** health promotion behaviors, intervention, multidimensional health, rural school students, school nurses, biology teacher

## Abstract

**Objective:**

Aim to explore the feasibility and preliminary impact of classroom-based and extracurricular interventions within eighth-grade biology curriculum to improve health promotion behavior (HPB) and multidimensional health of rural adolescents in China.

**Methods:**

An exploratory study with two surveys was conducted at eighth-grade students in a Chinese rural middle school. The intervention combined theoretical classroom instruction with practical extracurricular activities, all embedded within the biology curriculum. Given the incomplete pairing of the two survey samples, the data were analyzed as independent samples. Statistical significance was assessed using the Chi-square test for categorical variables and the Mann–Whitney *U* test for continuous variables.

**Results:**

Analyses indicated that the intervention was associated with significant positive outcomes. Compared with the first survey, the second had significantly higher overall HPB scores (*p* = 0.006, *r* = 0.155). Specifically, significant increases were observed in exercise behavior (*p* < 0.001, *r* = 0.231) and health responsibility (*p* = 0.004, *r* = 0.162). Additionally, the second survey group reported significantly better outcomes across multidimensional health, including self-reported health (*p* = 0.030, *r* = 0.121), physical health (*p* = 0.003, *r* = 0.167), and mental health (*p* = 0.009, *r* = 0.146). Subgroup analyses across both survey time points further revealed that the HPB-high group consistently scored significantly higher than the HPB-low group across all three health dimensions (all *p* < 0.001, *r* ≥ 0.460).

**Conclusion:**

This exploratory study confirmed the feasibility of classroom-based and extracurricular intervention and its potential to improve adolescent health. The observed correlation between high HPB and better health outcomes only suggests an association and the need for targeted support. Our findings recommend a collaborative model in which educators and health professionals jointly develop and implement interventions to advance the holistic development of rural adolescents.

## 1 Introduction

Developing healthy lifestyle behaviors has always been a priority for all age groups around the world (1). For adolescents, developing a healthy lifestyle is beneficial for various aspects of their health, such as physical, psychological, and cognitive health (2). Walker et al. (3) believes that health promoting behavior refers to a series of behaviors taken by individuals to prevent or detect diseases early, thereby realizing self-worth and improving or developing their own health status. Health promoting behavior can be used to help individuals, families, communities, and society to increase peace, happiness, to unleash their health potential (3). Schools are ideal places to carry out health promotion activities, and health promotion in schools is crucial for health and developing a healthy lifestyle among adolescents (4).

Adolescent children have increased ability for complex thinking, development of self-concept (5), but their concept and awareness of health promoting behavior are weak, they are prone to encountering health-related problems during their growth process (6). Many unhealthy habits developed in adolescence, such as imbalanced diet, lack of physical activity, poor mental state, high psychological pressure, and even violent behavior, which increase the risk of chronic diseases in adulthood (7). Depression, anxiety, and poor overall health-related quality of life are closely related to increased health risks (8). The physical activity deficiency rate of children and adolescents aged 6–17 in China is as high as 86.0%, and the overweight and obesity rate is 19% (9). In addition to gender, e.g., girls exercise less, and age (10), e.g., older adolescents are more likely to engage in risky behaviors, the health and healthy behavior of adolescents are also influenced by their socio-economic status, social relationships, and environment (11).

Improving adolescents’ concept and awareness of health promoting behavior requires health education (12). For rural middle school students, most parents work in other cities, and they stay at home with their grandparents on weekend and study at school from Monday to Friday, which means they have very little time with their parents (13). The acquisition of health knowledge and the formation of health promotion behaviors mainly come from school health education (14). Schools play a crucial role in helping adolescents filter out erroneous information by teaching students how to use reliable sources of information and promoting critical thinking through classroom learning, online dissemination, and extracurricular practical activities (15). In middle school biology textbooks, there are many learning resources for living a healthy life, fully reflecting that living a healthy life in biology teaching is not only a course requirement, but also has the advantages of classroom intervention in biology course (16). Classroom intervention refers to learning about health promoting behaviors in biology courses and applying them to the health practices of middle school students. Thus, biology teachers should deeply explore the content related to health promotion behaviors in textbooks in actual teaching activities (17), consciously and systematically integrate the cultivation of student health promoting behaviors into the classroom and beyond. Extracurricular intervention refers to organizing a series of extracurricular activities, such as themed speeches, expert reports, group discussions, watching pictures and videos, etc., to encourage middle school students to participate in health promotion activities to enhance their health literacy.

Therefore, this study adopted a two-survey design to explore feasible strategies associated with enhanced health outcomes among rural middle school students in China.

Specifically, this study was guided by the following questions:

*RQ1:* To what extent is the combined intervention (integrating classroom learning with extracurricular practice) associated with changes in the overall HPB and multidimensional health of rural middle school students?

*RQ2:* Were there significant differences in specific HPB dimensions and multidimensional health between the two surveys?

*RQ3:* This study examined whether significant differences existed between the high and low HPB groups in the two surveys, and whether heterogeneity was present across different health dimensions.

## 2 Methods

### 2.1 Study design

This study examined eighth-grade students at a rural middle school in Shangshui County, Zhoukou City, China, adopting two surveys and an eight-week intervention. The objective was to explore the feasibility of integrating HPB interventions into biology teaching for multidimensional health improving of rural middle students. The reason for selecting 8th grade students as participant is that the questionnaire survey was conducted during the eighth-grade biology course, which includes a chapter on “Living a healthy life”. The first survey was administered in October 2023; the intervention then commenced, and the second survey was conducted at the end of the program. In total, the two surveys and interventions lasted for approximately 8 weeks.

### 2.2 Participants and recruitment

Participants in this study were currently enrolled eighth-grade students from this rural middle school, voluntarily participated and provided signed informed consent. First, we conducted an online questionnaire survey in October 2023 (Appendices 1, 2) on Wenjuanxing software. Wenjuanxing software is a professional online survey, examination, evaluation, and voting platform that focus on providing users with a series of services, such as powerful and user-friendly online questionnaire design, data collection, custom reports, and survey result analysis. Then, all eighth-grade students were invited to complete the online questionnaire voluntarily. Ultimately, 222 students chose to participate and successfully submitted their responses. Participants with incomplete or unanswered responses to the questionnaire will be excluded. Finally, there were 179 (response rate: 80.63%) participants as valid participants. Subsequently, classroom learning and extracurricular practice interventions were carried out within the following 8 weeks. The 8-week intervention schedule can be found in Appendix 3. After interventions, the second questionnaire survey was conducted among 222 8th grade students who participated in the first survey. There were 141 (response rate: 63.51%) participants as valid participants in the second questionnaire survey with a decrease of 38 students (attrition rate: 21.23%).

Thus, the non-response rate was 19.37% for the first survey, primarily due to incomplete responses (43 students). The non-response rate for the second survey was 21.23%, mainly attributed to unwillingness to participate. Comparative analysis showed no significant differences in gender or age between respondents and non-respondents (*p* > 0.05), indicating minimal non-response bias (Table 1).

**Table 1:** Demographic characteristics.

Characteristics	The first survey (*n* = 179)		The second survey (*n* = 141)		*χ*^2^ (*Z*)	*p*
	Frequency	Proportion (%)	Frequency	Proportion (%)		
Gender					0.593	0.441
Male	89	49.7	64	45.4		
Female	90	50.3	77	54.6		
Age					1.381	0.167
	13.68 ± 0.613		13.74 ± 0.442			
Parental educational level					3.223	0.521
Junior high school and below	136	76.0	98	69.5		
Senior middle school	27	15.1	26	18.4		
Polytechnic school	8	4.5	8	5.7		
Junior college	3	1.7	6	4.3		
University/college/above	5	2.8	3	2.1		

To compare the characteristics between the first and second surveys (which were treated as independent due to incomplete pairing), Chi-square tests were used for categorical variables (e.g., gender and parental education level), and the Mann–Whitney *U* test was applied for the continuous variable (e.g., age).

The detailed Flow diagram of participant selection is shown in Appendix 4.

### 2.3 Intervention strategies

The intervention integrates classroom learning and extracurricular practice. Designed and developed under the joint guidance of biology teachers and school nurses, it was implemented by biology teachers among all eighth-grade students. All biology teachers adopted standardized textbooks, teaching content, and instructional approaches when delivering the “Living a Healthy Life” chapter in the eighth-grade biology curriculum. Teachers facilitated student discussions on questions such as “What do you think health is?”, “What is a healthy life, and how does lifestyle affect health?”, and “Do you think your lifestyle is healthy?” to introduce the lesson objectives and content. The classroom learning intervention was divided into the following seven steps and carried out over 3 weeks (Table 2). There was one class per week, each lasting 45 min. Each class had approximately 50 students, with a total of 280 students across all classes.

**Table 2:** Classroom learning intervention strategy of choosing a healthy lifestyle.

Items and contents
Creating context and introducing a new lesson
Display several unhealthy lifestyles: high-fat, high sugar, high salt diets, smoking, alcoholism, etc. Guide students to think, “What do you think the above lifestyles have on our health?”
Introduce the concept of healthy lifestyle.
Autonomous learning and discovering problems
Student self-directed learning lifestyle disease, e.g., chronic, and non-communicable diseases such as cardiovascular disease, cerebrovascular disease, malignant tumors, etc.
The teacher explains what lifestyle diseases are and the relationship between lifestyles and health.
Collaborative learning and analyzing problems
Group discuss the impact of lifestyle on health, and the hazards of unhealthy lifestyles.
Discuss whether one’s own and family’s lifestyle is healthy.
Collaborative learning and solving problems
Let students write their healthy and unhealthy lifestyle behaviors.
Let the students discuss and exchange ideas and draw a “health tree”.
The groups sent representatives to summarize the content of healthy lifestyles, and other students made corrections, supplements, and improvements.
Make students aware that a healthy lifestyle should be cultivated from an early age.
Integrating and applying knowledge what is learned
Using facts to make students aware of the harm caused by unhealthy lifestyles.
Discuss how to maintain a healthy lifestyle in the future?
Summary, consolidation, and improvement
Guide students to talk about the gains and confusions from this lesson.
Then organize students to review and summarize the content learned in this lesson.
Reflect on this lesson to form your own knowledge system.
After class practice and knowledge implementation
Summarize several methods for developing a healthy lifestyle.
Design your own three meals a day based on healthy lifestyles.

Extracurricular practices were conducted in two ways: the “Healthy Life Theme Class Meeting” and “Adolescent Classes” (Table 3). The healthy lifestyle themed class meeting was held every 2 weeks under the theme “A Better Life, Starting with a Healthy Lifestyle.” The theme was selected based on biology learning content and students’ unhealthy lifestyle behaviors. First, biology teachers and school nurses guided students to talk about their own lifestyles and discuss whether their lifestyles were healthy, as well as questions such as “How can we cultivate a healthy lifestyle?” and “How important is a healthy lifestyle for teenagers?” After the student discussion, the teachers and nurses guided them to write down their experiences and plans for cultivating healthy lifestyles in the future. This themed class meeting was promoted through sharing, communication, and interaction. Second, biology teachers and school nurses gathered all the students together to deliver a keynote speech and present cases of maintaining a healthy lifestyle, aiming to enhance students’ understanding of the importance and necessity of healthy lifestyles among teenagers. Due to the particularities and differences of puberty between boys and girls, the Adolescent Classes followed the steps outlined in Table 3, with boys and girls learning separately.

**Table 3:** Extracurricular intervention strategies.

Items and contents
Theme Class Meeting (boys and girls learn and discuss together).
The biology teachers and school nurses guided students to discuss healthy lifestyles freely.
The students wrote down experiences and plans for future healthy lifestyles.
The biology teachers and school nurses gave a keynote speech for all the students.
Adolescent Classes (boys and girls learn puberty knowledge separately).
The biology teachers and school nurses:
Explained to students the changes experienced by adolescents.
Imparted health care knowledge in the form of question chains.
Taught them how to take care of their health during adolescence.
Listed some life examples and analyzed the secrets to maintaining good emotions.
Guided adolescent students to establish a correct concept of life and health.

### 2.4 Measures

#### 2.4.1 Health promotion behaviors

The HPB of rural middle school students includes six dimensions (Appendix 1): exercise behavior, dietary and nutritional behavior, health responsibility, interpersonal relationship, stress management, life appreciation (18). Each dimension contains five questions, and each measured using a 5-point Likert scale, with a value from 1 for “strongly disagree” to 5 for “strongly agree.” The score for each dimension is between 5 and 25. The higher score indicates the better HPB.

#### 2.4.2 Multidimensional health

The multidimensional health of rural middle school students was evaluated from three dimensions (Appendix 2) of self-reported health, physical health, and mental health (19, 20). Each dimension was evaluated using one question, and all three questions are measured using a 5-point Likert scale. The higher score indicates the more health.

#### 2.4.3 Data analysis

The data used in this study were derived from two surveys. Given that the surveys were anonymous and individual participants could not be matched, the data were analyzed as independent samples. Mann–Whitney *U* test was performed using IBM SPSS Version 20 (IBM, Armonk, NY, United States) to assess continuous variable(e.g., age, HBP, and Multidimensional health) differences between the first and second surveys, as well as using the Chi-square test for categorical variables (e.g., gender and parental education level). Descriptive statistics [mean, standard deviation (SD), frequency and proportion] provide an overview of the sample group, including gender, age, and parental education level.

#### 2.4.4 Psychometric properties of the questionnaires

To ensure the psychometric quality of the instrument, its validity was assessed, with the results detailed in Table 4. All Cronbach’s alpha values are greater than 0.8, indicating excellent reliability and validity.

**Table 4:** Psychometric properties of the questionnaires.

Items	Cronbach’s alpha	
	The first survey (*n* = 179)	The second survey (*n* = 141)
Exercise behavior	0.871	0.893
Nutritional behavior	0.889	0.877
Health responsibility	0878	0.897
Interpersonal relationship	0.895	0.921
Stress management	0.906	0.931
Life appreciation	0.940	0.955
Total	0.968	0.955
Multidimensional health	0.804	0.850

## 3 Results

### 3.1 Demographic characteristics

As shown in Table 1, there are 179 students (male: 49.7%, female: 50.3%) participated in the first survey, and 141 students (male: 45.4%, female: 54.6%) participated in the second survey. Approximately 70% (the first survey: 76%, the second survey: 69.5%) of parents have a junior high school and below education level, indicating a lower level of education.

### 3.2 Comparison of HPB differences between the first and second surveys

**Figure 1:**
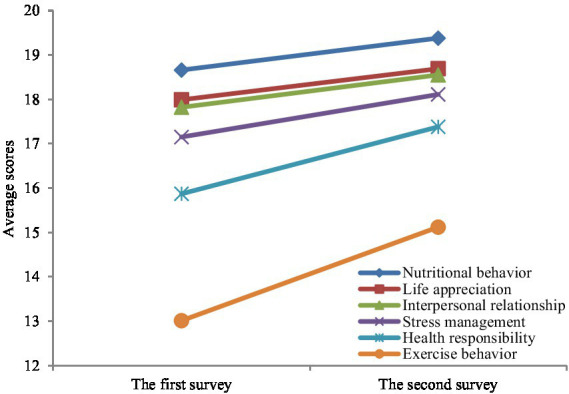
HPB differences between the first and second surveys.

Figure 1 showed that the HPB of the rural middle school students had a significant improvement in the second survey compared with the first survey (detailed in Appendix 5), with the total score increasing from 100.50 ± 25.520 to 107.22 ± 25.770, and the difference was statistically significant (*p* = 0.006, *r* = 0.155). Specifically, dimensions of exercise behavior (*p* < 0.001, *r* = 0.231) and health responsibility (*p* = 0.004, *r* = 0.162) exhibited significantly increases. Nutritional behavior had the highest scores among all dimensions, but no statistically significant difference was observed (*p* = 0.120, *r* = 0.087), as well as the dimensions of life appreciation, interpersonal relationship, and stress management with minor increases (*p* > 0.05). Thus, the classroom-based and extracurricular intervention was significantly associated with improvements in exercise behavior and health responsibility among rural middle school students.

### 3.3 Comparison of multidimensional health differences between the first and second surveys

Multidimensional health differences between the first and second surveys were performed using the Mann–Whitney *U* test. Effect sizes (*r*) were calculated as *r* = *Z*/√*N* to quantify the magnitude and practical significance of the observed differences. As shown in Table 5, significant differences were observed between the two surveys across all three dimensions of health. Specifically, respondents in the second survey reported significantly higher scores than those in the first survey for self-reported health (*p* = 0.030, *r* = 0.121), physical health (*p* = 0.003, *r* = 0.167), and mental health (*p* = 0.009, *r* = 0.146). Among these, physical health exhibited the largest effect size (*r* = 0.167), indicating a relatively stronger association across the two surveys, while mental health showed the highest mean score in the second survey (M = 4.01, SD = 1.146). These findings suggest that respondents in the second survey reported better multidimensional health compared with the first survey.

**Table 5:** Multidimensional health differences between the first and second surveys.

Multidimensional health	The first survey (*n* = 179)	The second survey (*n* = 141)	*U*	*Z*	*p*	*r*
Self-reported health	3.60 ± 0.974	3.82 ± 0.995	14319.000	2.173	0.030	0.121
Physical health	3.22 ± 1.082	3.56 ± 1.051	14974.500	2.981	0.003	0.167
Mental health	3.68 ± 1.215	4.01 ± 1.146	14657.000	2.603	0.009	0.146

The results were expressed as Mean ± SD. *p* values were calculated by Mann–Whitney *U* test.

### 3.4 Comparison of multidimensional health differences among HPB groups

**Figure 2:**
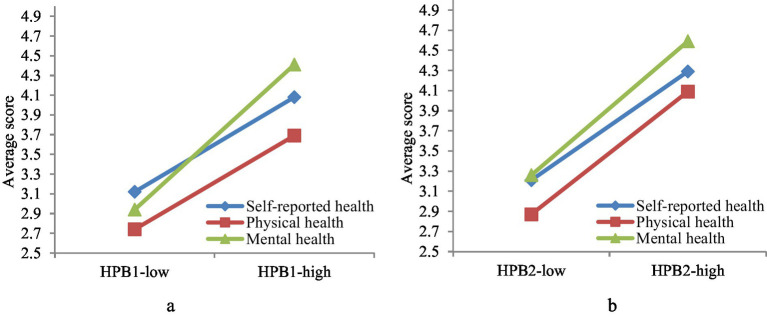
Multidimensional health differences among high and low HPB groups. **(a)** HPB1 groups. **(b)** HPB2 groups.

Figures 2a,b showed that the high HPB group scored significantly higher than the low HPB group across all three dimensions in the two surveys (detailed in Appendix 6): self-reported health, physical health and mental health (*p* < 0.001). While the health status of both groups improved in the second survey compared with the first survey, the disparity between the groups exhibited diverging trends. In the physical health dimension, the difference between the high and low groups widened from 0.95 to 1.22, indicating that individuals with high health behaviors possess greater advantages or potential for improving physiological function. The high HPB group consistently maintained extremely high scores in the mental health dimension (from 4.41 up to 4.59), far surpassing the low group. This indicates that HPB play an important role in psychological well-being. Elevating the HPB levels among rural middle school students, especially through targeted interventions for the low HPB group, is essential for comprehensive health improvement.

## 4 Discussion

### 4.1 Main findings

Our findings suggest that integrating classroom instruction with extracurricular practice appears to be a viable strategy for improving rural student health. Specifically, this curriculum-based strategy yielded notable improvements in HPB among rural eighth-grade students, particularly in exercise behavior and health responsibility. Compared with the first survey, multiple multidimensional health indicators, namely self-reported health, physical health, and mental health, also presented statistically positive changes. Moreover, across two surveys, the high HPB group consistently scored significantly higher than the low HPB group in all health dimensions. While the observed effect size was relatively minor, the findings still offer valuable implications for the design and implementation of school health promotion programs.

Due to the significant diversity in intervention design and methods, target groups, duration, funding, and evaluation design of school health promotion projects, comparing our results with other research findings is limited. However, many countries and regions around the world have already carried out health promotion education at different levels. For a high school in Hungary, a comprehensive health promotion program showed that after 6 months of student intervention, girls’ self-rated health status was significantly improved compared to boys, with a significant decrease in consumption of sweets and sugary soft drinks, but a significant increase in the proportion of girls lacking physical activity (21). The preliminary study showed that female students have a higher level of health promoting behavior than male students, with significant gender differences only in health responsibility behavior and interpersonal relationship dimensions (22). An evaluation was conducted on six health behaviors among adolescents in northern Saudi Arabia, including nutrition, social support, health responsibility, life appreciation, exercise, and stress management. It was found that over half (52.7%) of health promoting behaviors were lower and significantly correlated with gender. It is feasible to improve HPB by developing awareness raising and health promotion intervention plans for adolescents (23).

The alcohol intervention for young people in rural Australia had reduced their risk of drinking culture, and nutrition intervention programs in Australian secondary schools can effectively improve nutrition related health outcomes (24). The health literacy and health promotion behaviors of adolescents in Turkey can improve the health literacy by incorporating basic health knowledge and healthy lifestyle behavior courses into the student curriculum (25). The 2010 Washington State Health Youth Survey found that mental health status is closely related to the number of behaviors recognized by adolescents to promote health. In adolescent health initiatives in schools, communities, and primary healthcare, interventions that increase the use of sleep hygiene and exercise habits should be added to a comprehensive and proactive youth development framework (26). John et al. (27) demonstrated that a school-based intervention conducted in India is feasible and preliminarily effective in improving adolescents’ health cognition, including health knowledge, self-efficacy, behavioral intentions, and health locus of control. In short, schools across diverse countries and regions have explored student health promotion and achieved promising outcomes. Ample evidence confirms that well-designed interventions can effectively improve adolescents’ HPB and health. Moreover, the findings of this study can be generalized to comparable rural schools with limited health support resources worldwide.

### 4.2 Intervention design: reflections and future directions

Adolescence is a critical period for cultivating healthy lifestyles, characterized by rapid physiological maturation that often outpaces psychological development (28). This non-equilibrium state, compounded by increasing academic pressure and complex interpersonal dynamics, renders adolescents particularly vulnerable to psychological distress, such as anxiety and depressive symptoms (29). Furthermore, peer influence (30) often drives unhealthy dietary choices (e.g., high-sugar beverages and fried foods) (31)and physical inactivity, increasing the risk of obesity (32). Adolescent girls face additional physiological challenges (33); the onset of menstruation and rapid growth significantly elevate iron requirements, making them highly susceptible to iron-deficiency anemia (34). These challenges are further intensified among students in rural boarding schools, who often lack sufficient family care, thereby rendering the supportive roles of teachers and school nurses indispensable. However, empirical research on health promotion interventions tailored for this specific rural population remains notably scarce. To bridge this gap, our study leveraged existing educational resources. Specifically, the health-related chapters in the middle school biology curriculum through a collaborative effort between biology teachers and school nurses.

Integrating classroom teaching with extracurricular activities to enhance rural middle school students’ HPB and comprehensive health represents advancement over traditional teaching approaches. It not only sparked students’ interest in health-related biology content but also allowed them to consolidate knowledge through practical application. Given that adolescent psychological issues are predominantly rooted in a lack of emotional regulation skill, which subsequently impact physical health, the intervention specifically incorporated modules on emotional management (35). Ultimately, by grounding instructional design in real-life contexts (36), the initiative aimed to deepen students’ intuitive understanding of health, clarify the associations between HPB and multidimensional health outcomes, and enable students to gradually reduce unhealthy habits and adopt sustainable healthy lifestyles.

### 4.3 Limitations

Several limitations of this study should be acknowledged. First, the cross-sectional design precludes causal inferences between variables. Although significant associations were observed between the interventions and health outcomes, the findings reflect correlational relationships at a single time point rather than causal effects. Future research should adopt longitudinal designs or randomized controlled trials to validate these findings and clarify their causal mechanisms. Second, incomplete pairing between the two surveys means that bias may still exist. To address this limitation, participants’ demographic characteristics were compared and no significant differences were found. Although obvious selection differences were absent, and potential bias may still exist, which nevertheless supports the rationality of regarding each group as an independent sample. Furthermore, systematic disparities between dropouts and retained participants may induce Type I error, while sample attrition reduces statistical power and heightens the risk of Type II error. Third, the single-school design, limited sample size, and absence of a parallel control group restrict causal inference and reduce the generalizability of the findings to diverse international settings worldwide. Notwithstanding these limitations, the distinct differences in HPB and comprehensive health observed among rural students provide a valuable reference for designing and implementing future health intervention strategies worldwide.

## 5 Conclusion

The findings indicate that combining biology classroom teaching with extracurricular intervention practices is a feasible strategy significantly associated with health improvement among rural middle school students. The significant associations among exercise, health responsibility, and multidimensional health indicate that embedding health promotion into biology education may help translate classroom knowledge into tangible well-being outcomes for rural students. Furthermore, the good health outcomes observed in the HPB-high group underscore the importance of targeted behavioral support for students with lower HPB levels. While teachers are ideally positioned to deliver health content, they often require support in designing targeted behavioral interventions. School nurses should act as expert consultants, equipping teachers with evidence-based strategies and practical resources. This Teacher-Nurse Synergy model effectively integrates pedagogical instruction with clinical health expertise. With limited daily family care and supervision, rural boarding students are particularly vulnerable to health risks. This comprehensive model provides a crucial compensatory health support strategy with broad implications for global rural school health promotion.

## Data Availability

The original contributions presented in the study are included in the article/Supplementary material, further inquiries can be directed to the corresponding author.
