# Supplementary material for: Integrating health promotion into biology education: effects of classroom and extracurricular interventions in rural adolescents
Source: Front Nutr. 2026 May 28;13:1813900. doi: 10.3389/fnut.2026.1813900 (PMC13253422; doi:10.3389/fnut.2026.1813900)
Supplement: Supplementary file 1 [file Table_1.DOCX]

Appendix 1 Health promotion behavior scale.

| Items | |
| --- | --- |
| 1.Exercise behavior | |
| Following a well-designed exercise plan. | |
| At least three intense (sweating) exercises per week (for more than 20 minutes). | |
| Doing physical exercise from daily life (such as taking a walk after meals, taking fewer cars, and walking more). | |
| Participating in some entertainment activities (such as swimming, dancing, cycling). | |
| I measure my pulse during or after exercise. | |
| 2. Dietary and nutritional behavior | |
| Eating breakfast every day. | |
| Eating rice, noodles, and grains every day. | |
| Eating meat, poultry, fish, dried beans, eggs, and nuts every day. | |
| Eating fiber rich foods every day (such as fruits, vegetables, etc.). | |
| Reading the labels of packaged food, check the production date, shelf life, and precautions. | |
| 3.Health responsibility behavior | |
| When coughing or sneezing, it will cover the mouth and nose. | |
| Seeking timely medical attention for health issues and seeking guidance and advice if necessary. | |
| Reading or watching books or TV programs that promoting health, and learn about health knowledge and skills. | |
| Participating in health education activities. | 5- Likert scale:   - There are a total of six dimensions, each containing five questions. - Each question ranges from 1 for “strongly disagree” to 5 for “strongly agree.” - Each question score varies between 1 and 5. - Each dimension score varies between 5 and 25. - The higher score indicates the more health promotion behavior. |
| Self-checking your body at least once a month. |  |
| 4. Interpersonal relationship behavior |  |
| Proactively helping those in need and provide care, love, and warmth. |  |
| Maintaining meaningful interpersonal relationships. |  |
| Making time to engage in activities or discuss issues with family and friends. |  |
| Actively communicating or confide in others when facing troubles. |  |
| Willing to praise others for their success. |  |
| 5. Stress management behavior |  |
| Finding some time every day to relax you. |  |
| Being able to accept things in life that one cannot change |  |
| I will pay attention to my emotional fluctuations and actively adjust. |  |
| Searching for suitable methods to relieve stress. |  |
| Thinking of something happy before bedtime. |  |
| 6. Life appreciation behavior |  |
| Believing that one’s life is purposeful and constantly striving for it. |  |
| Feeling one actively growing and changing. |  |
| Knowing what is important to you in life. |  |
| Willing to accept new experiences or challenges. |  |
| Full of expectations for the future. |  |

Note. Appendix 1 was referenced from the literature (Wang et al., 2009) with revision.

Appendix 2 Multidimensional health scale.

| Items | 5- Likert scale |
| --- | --- |
| 1. Are you currently in good health? | - Three dimensions and each containing one question. - Each question ranges from 1 for “strongly disagree” to 5 for “strongly agree.” - The higher score indicates the more health. |
| 1. Do you feel energetic? |  |
| 1. Are you full of hope for your future life? |  |

Note. Appendix 2 was referenced from the literature (Tian & Chen, 2022; Sharma et al., 2016) with revision.

Appendix 3 An 8-week intervention schedule

| Items | Weeks | Contents | Notes |
| --- | --- | --- | --- |
| 1 | 1 | The first survey | Online questionnaire |
| 2 | 2-4 | Classroom learning intervention | Classroom teaching |
| 3 | 4-5 | Extracurricular interventionⅠ: Theme Class Meeting | Multiple times in spare time |
|  | 6-7 | Extracurricular interventionⅡ: Adolescent Classes | Multiple times in spare time |
| 4 | 8 | The second survey | Online questionnaire |

Appendix 4 Flow diagram of participant selection


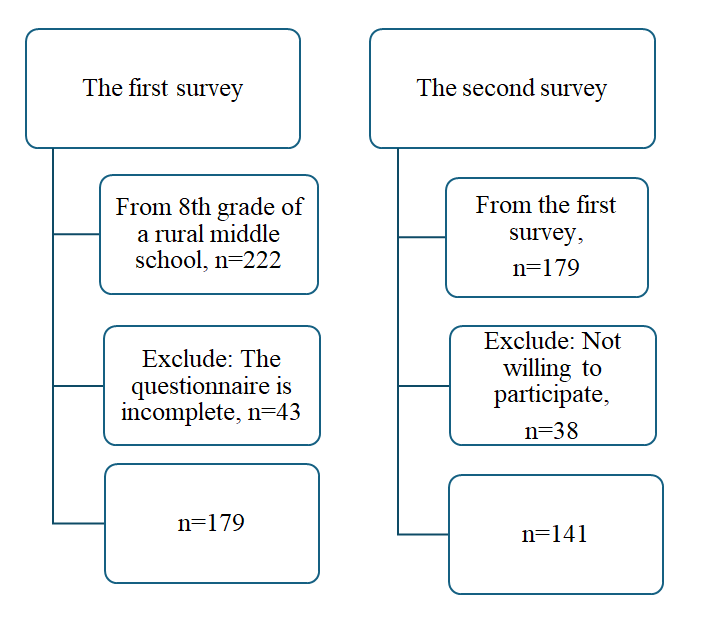


Appendix 5 Health promotion behavior differences between the first and second surveys

| Characteristics | The first survey  (n = 179) | The second survey  (n = 141) | U | Z | P | r |
| --- | --- | --- | --- | --- | --- | --- |
| Exercise behavior | 13.01 ± 4.586 | 15.12 ± 4.856 | 15999.500 | 4.124 | ˂ 0.001 | 0.231 |
| Nutritional behavior | 18.66 ± 4.578 | 19.38 ± 4.404 | 13891.000 | 1.554 | 0.120 | 0.087 |
| Health responsibility | 15.87 ± 5.073 | 17.38 ± 5.194 | 14988.000 | 2.889 | 0.004 | 0.162 |
| Interpersonal relationship | 17.82 ± 5.030 | 18.55 ± 4.816 | 13753.000 | 1.385 | 0.166 | 0.077 |
| Stress management | 17.15 ± 5.082 | 18.11 ± 5.117 | 14070.000 | 1.773 | 0.076 | 0.099 |
| Life appreciation | 17.99 ± 5.424 | 18.69 ± 5.226 | 13669.000 | 1.286 | 0.199 | 0.072 |
| Total | 100.50 ± 25.520 | 107.22 ± 25.770 | 14897.500 | 2.773 | 0.006 | 0.155 |

Note. The results were expressed as Mean±SD. P values were calculated by Mann-Whitney U test.

Appendix 6 Multidimensional health differences among health promotion behavior levels

|  | The first survey (n=179) | |  |  |  |  |
| --- | --- | --- | --- | --- | --- | --- |
| Multidimensional health | HPB1-low | HPB1-high | U | Z | P | r |
|  | ＜100.50 | ≥100.5 |  |  |  |  |
| Self-reported health | 3.12 ± 0.939 | 4.08 ± 0.753 | 6221.000 | 6.739 | ˂ 0.001 | 0.504 |
| Physical health | 2.74 ± 0.936 | 3.69 ± 1.013 | 6048.500 | 6.152 | ˂ 0.001 | 0.460 |
| Mental health | 2.94 ± 1.080 | 4.41 ± 0.847 | 6774.500 | 8.325 | ˂ 0.001 | 0.622 |
| Multidimensional health | The second survey (n=141) | | U | Z | P | r |
|  | HPB2-low | HPB2-high |  |  |  |  |
|  | ＜107.22 | ≥107.22 |  |  |  |  |
| Self-reported health | 3.21 ± 0.951 | 4.29 ± 0.750 | 3925.000 | 6.492 | ˂ 0.001 | 0.547 |
| Physical health | 2.87 ± 0.991 | 4.09 ± 0.750 | 4009.000 | 6.818 | ˂ 0.001 | 0.574 |
| Mental health | 3.26 ± 1.223 | 4.59 ± 0.650 | 3962.000 | 6.767 | ˂ 0.001 | 0.570 |

Note. HPB is the abbreviation for health promotion behavior. HPB1 and HPB2 stand for total health promotion behavior in the first and second survey, respectively. HPB1-low and HPB1-high stand for the low and high group of health promotion behavior in the first survey. Similarly, HPB2-low and HPB2-high stand for the low and high group of health promotion behavior in the second survey. The results were expressed as Mean ± SD. P values were calculated by Mann-Whitney U test. The numbers 100.5 and 107.22 are the average scores of total health promoting behaviors in the first and second surveys, respectively, as cutoff scores for high and low HPB groups.
